# Implementing disability evaluation and welfare services based on the framework of the international classification of functioning, disability and health: experiences in Taiwan

**DOI:** 10.1186/1472-6963-13-416

**Published:** 2013-10-14

**Authors:** Wen-Ta Chiu, Chia-Feng Yen, Sue-Wen Teng, Hua-Fang Liao, Kwang-Hwa Chang, Wen-Chou Chi, Yen-Ho Wang, Tsan-Hon Liou

**Affiliations:** 1 Ministry of Health and Welfare, No. 36, Tacheng Street, Datong District, Taipei City 10341, Taiwan; 2 Graduate Institute of Injury Prevention and Control, Taipei Medical University, No. 250 Wu-Hsing Street, Taipei 11031, Taiwan; 3 Department of Public Health, Tzu Chi University, Hualien, No. 701, Sec. 3, Jhongyang Road, Hualien City 97004, Taiwan; 4 Chinese Association of Early Intervention, Profession for Children with Developmental Delays, No. 63, Kuofu Street, Hualien City 97004, Taiwan; 5 School and Graduate Institute of Physical Therapy, College of Medicine, National Taiwan University, 3rd floor, No 17, Hsuzhou Road, Taipei 10055, Taiwan; 6 Department of Physical Medicine and Rehabilitation, Wan Fang Hospital, Taipei Medical University, NO. 111, Section 3, Hsing-Long Road, Taipei 11696, Taiwan; 7 Department of Information Management, National Chung Cheng University, No. 168, Sec. 1, University Road, Min-Hsiung Township, Chia-yi County 62102, Taiwan; 8 Department of Physical Medicine and Rehabilitation, National Taiwan University Hospital, National Taiwan University, No. 1, Changde Street, Zhongzheng District, Taipei City 10048, Taiwan; 9 Department of Physical Medicine and Rehabilitation, Shuang Ho Hospital, Taipei Medical University, No. 291, Zhongzheng Road, Zhonghe District, New Taipei City 23561, Taiwan

**Keywords:** International classification of functioning, Disability and health (ICF), Disability evaluation, World Health Organization (WHO)

## Abstract

**Background:**

Before 2007, the disability evaluation was based on the medical model in Taiwan. According to the People with Disabilities Rights Protection Act, from 2012 the assessment of a person’s eligibility for disability benefits has to be determined based on the International Classification of Functioning, Disability, and Health (ICF) framework nationwide. The purposes of this study were to: 1) design the evaluation tools for disability eligibility system based on the ICF/ICF-Children and Youth; 2) compare the differences of grades of disability between the old and new evaluation systems; 3) analyse the outcome of the new disability evaluation system.

**Methods:**

To develop evaluation tools and procedure for disability determination, we formed an implementation taskforce, including 199 professional experts, and conducted a small-scale field trial to examine the feasibility of evaluation tools in Phase I. To refine the evaluation tools and process and to compare the difference of the grades of disability between new and old systems, 7,329 persons with disabilities were randomly recruited in a national population-based study in Phase II. To implement the new system smoothly and understand the impact of the new system, the collaboration mechanism was established and data of 168,052 persons who applied for the disability benefits was extracted from the information system and analysed in Phase III.

**Results:**

The measures of the 43 categories for body function/structure components, the Functioning Scale of Disability Evaluation System for activities/participation components, and the needs assessment have been developed and used in the field after several revisions. In Phase II, there was 49.7% agreement of disability grades between the old and new systems. In Phase III, 110,667 persons with a disability received their welfare services through the new system. Among them, 77% received basic social welfare support, 89% financial support, 24% allowance for assistive technology, 7% caregiver support, 8% nursing care and rehabilitation services at home, and 47% were issued parking permits for persons with disability.

**Conclusion:**

This study demonstrated that disability evaluation system based on the ICF could provide a common language between disability assessment, needs assessment and welfare services. However, the proposed assessment protocol and tools require additional testing and validation.

## Background

**Figure 1:**
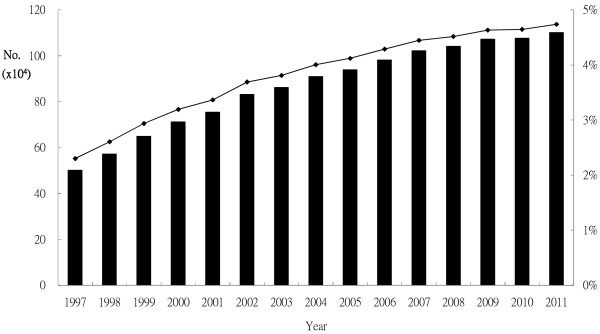
**Trend and prevalence rates of disability in Taiwan.** The trend and estimated prevalence rates for disability in Taiwan between 1997 and 2011. Black bars indicate the number of people with disability each year, and the line shows the percentage of people with disability among the general population each year.

Disability may be defined as negative interactions between the functional impairments of people and their environment, with problematic consequences [1]. People with disabilities often experience difficulties in their daily lives, and are limited in their activities and social participation, and comprise 15% of the global population with a minimum estimated 1 billion [2]. The number of people with disabilities increased every year in Taiwan from 3.8% in 2003 to 4.6% in 2011 [3] (Figure 1).

**Figure 2:**
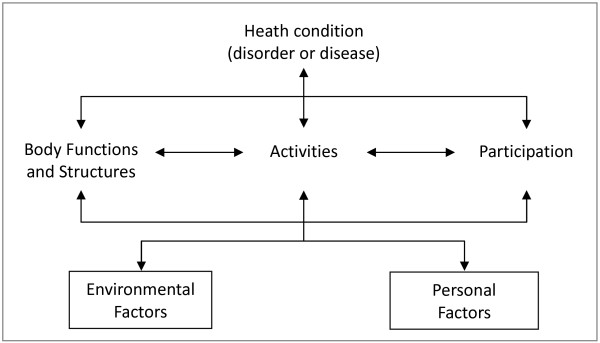
The Framework of International Classification of Functioning, Disability, and Health (ICF) and interactions between the components.

For decades, the definition of disability remained unclear and debated among experts in the medical and social sciences [1]. However, two major advances have occurred in disability research recently: the International Classification of Functioning, Disability, and Health (ICF) and the United Nations Convention on the Rights of Persons with Disabilities (UN CRPD). The first is the promulgation of the ICF in 2001 by the World Health Organization (WHO) [4]. Traditionally, disabled people have been viewed from a medical perspective. Disability had been narrowly equated with the health condition, impairment, or capacity limitations of people. This overly medicalized view fails to address the social factors, discrimination, prejudice, and barrier of environmental factors that prevent the full participation of people with disabilities, and unable to describe the factors, such as assistive technology, that contribute to the overall disability experience [4,5]. The ICF framework was developed by the WHO to describe health and disability at both the individual and societal level (Figure 2). The ICF framework assists in examining an individual functioning at the physical, personal, and societal levels, and provides definitions to conduct operational assessments. The ICF system provides a universal framework for assessing the functioning of any person. The ICF classifications are based on the understanding that for any person, various factors interact, and all these factors must be considered to perform a proper assessment; hence, several components are included, such as body function and structure, activities and participation, and environmental and personal factors (Figure 2). The ICF system provides an excellent scientific approach to collecting reliable statistics on disabled populations. In Japan, Italy, and Australia, and Portugal, the ICF/ICF-Children and Youth version (ICF-CY) framework has been used to guide clinical measurements and evaluations of people requiring rehabilitation, home care, elderly adult care, special education, and disability support [4,6-8]. The ICF framework was also used in the Multi-Country Survey Study conducted by the WHO between 2000 and 2001, and the World Health Survey Program of 2002 and 2003, to measure the health status of general populations in 71 countries [4].

Second, the UN CRPD was adopted by the United Nations in December 2006, and it became effective in May 2008. People with disabilities often require special support in various areas such as education, housing, work, and social benefits to assist them to live and participate in their community. The CRPD states that all mainstream health services are inclusive of people with disabilities, especially for older adults, women, and people with a low economic status.

Since 1980, the Taiwanese government enacted certain legislative procedures to create and revise categories regarding disabilities. A person who fulfills the eligibility criteria for disability benefits may be granted financial aid and in-kind benefits from the government. However, the criteria for disability evaluation were mainly based on the medical model that considered disability as a physical and mental impairment before 2007. Thus, physicians identified candidates for disability benefits based mainly on their severity of body impairment, but without a sufficient evaluation of their daily activity, participation, and environmental factors. In 2007, Taiwan legislated a constitutional amendment known as the People with Disabilities Rights Protection Act [9]. Since July 2012, the act has mandated that the assessment of individual eligibility for disability benefits should be based on the ICF framework. The purpose of this new national-level model is to form links among disability evaluation, needs assessment and social welfare services, in order to promote the participation of people with disabilities.

To prepare the new disability eligibility system, the Taiwanese government authorized professionals to form a Taiwanese ICF taskforce. The taskforce missions were to develop standardized measures and regulations of disability evaluation and to monitor the impacts of the new eligibility system. The specific aims of this study were to: 1) design the evaluation tools for disability eligibility system based on the ICF and ICF-child and youth (ICF-CY); 2) compare the differences of disability grades between the old and new systems; 3) analyse the outcome of the new disability evaluation system.

## Methods

**Figure 3:**
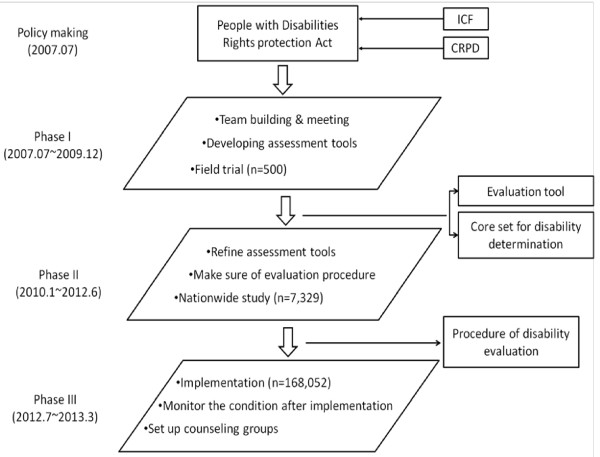
**Development of the new disability evaluation and welfare services in Taiwan.** Abbreviation: ICF: International Classification of Functioning, Disability, and Health; CRPD: Convention on the Rights of People with Disability.

The preparation for the reformation of the disability system began in 2007 by Taiwan authorities of health and social welfare in collaboration with researchers and social welfare groups. The activities of three main phases to reach the specific aims are shown in Figure 3 and described in the following sections. We applied to the registration system, Minister of Interior, Taiwan and got this anonymized data. The Joint Institutional Review Board at the Taipei Medical University approved this study (approval number of 201004001 and 201205042).

### Phase I (July 2007 to December 2009): evaluation tools development

We formed an implementation taskforce for this reform to survey existing resources and measures tools for people with disabilities, to review the literature, to design measurement items based on the selected ICF/ICF-CY categories, and to conduct a small-scale field trial to examine the feasibility of the developed evaluation tools.

#### Step 1: taskforce building and meeting

From June 2007 to December 2009, the health team facilitated 16 focus groups that were attended by 199 professional experts. These members included physicians, dentists, nurses, physical therapists, occupational therapists, social workers, psychologists, special education teachers, vocational assessment workers, public health scholars, and representatives of welfare groups for people with disabilities. All of them were familiar with the ICF and/or with disability assessments. Eight groups focused on the ICF components of body functions and body structures (b/s), and 8 groups focused on the ICF components of activities and participation (d) and environmental factors (e). Each focus group included 5 to 10 experts, and meetings were held periodically. A leader was appointed in each group to lead the discussions and assist the group in reaching an agreement through a consensus or by a vote. Several leader group meetings were also held to decide on the following principles: (1) Measure items are designed based on second level categories of ICF/ICF-CY; (2) Both ICF and ICF- CY categories should be considered; (3) Scale of each item is designed based on generic qualifiers for b, s, and d categories (0 represented no problem, and 4 represented complete problem), and binary scale for e categories (either as having a barrier or no barrier); (4) Assessment should be based on existing tools with sound psychometrics, such as the Wechsler Adult Intelligence Scale third edition (WAIS-III) for intelligence assessment and the Berg Balance Scale for balance evaluation.

For the needs assessment of social welfare services, the social welfare authority assembled another professional group simultaneously. The group was tasked to develop the needs assessment tool for people with disabilities, to check the services at the local government for stakeholders’ take, and to develop the training materials for testers to conduct needs assessments based on references related to the ICF/ICF-CY.

#### Step 2: developing assessment tools for medical and functional assessments

In the 8 focus groups on body functions and body structures (b/s), each group selected categories for disability evaluation through the participation of several medical associations by numerous meeting and electronic mails. The standardized examination methods for each category, including the rating scale to assign qualifiers, were also determined and adjusted through the physicians’ previous disability evaluation and clinical experiences, consensus meetings and empirical data analyses of pilot studies.

In the 8 focus groups on activities and participation and environmental factors (d/e), measures for the 137 categories that were selected from the ICF checklist by consensus meeting were designed for the trial version of functional assessment [10,11]. An operation manual of the trial version which included standardized testing methods and qualifiers rating was drafted. However, due to practical reasons and the lengthy time required to administer the trial version, the d/e focus groups later decided to adopt the 36-item version of the WHO Disability Assessment Schedule 2.0 (WHODAS 2.0) [12] instead of the trial version [10,11] in the performance dimension of the Functioning Scale of the Disability Evaluation System-Adult Version (FUNDES-adult version). The advantage of the WHODAS 2.0 is that it can be used cross-culturally and has been tested in more than 10 countries [13].

Between 2007 and 2012, all of the groups held 57 meetings to discuss the disability evaluation (22 meetings for the ICF components b and s, and 35 meetings for the ICF components d and e); 19 meetings were for the group leaders; and 75 meetings focused on the ICF-CY version. The meetings were held across Taiwan.

#### Step 3: developing measurement tools for needs assessment

For understanding the profile of welfare resources and developing the needs assessment tool, a resource checklist and a structured questionnaire were mailed to the administrators in charge of welfare services in every city in Taiwan. The needs assessment tool was also based on the ICF d component and the possible environmental barriers.

#### Step 4: a small-scale field trial

A small-scale field trial was conducted in 2009 to test the feasibility of the trial version of disability evaluation. Several testers (registered therapists in the authorized hospitals) were trained by 4 training programs in two cities and two counties to collect data. A total of 500 persons with disabilities in those 4 cities or counties participated in this trial.

### Phase II (January 2010-June 2012): evaluation tools refinement and validation

#### Step 5: refine evaluation tools

After the small-scale field trial, the 18 focus groups for b/s/d continued to refine the tools throughout the meetings according to previous trial experiences and data analyses. For the b/s assessment and functional assessment tools, the first priority was to develop a core set for disability evaluation. The main references for the core set included the manuals of the ICF [4] /ICF-CY [5], the WHODAS 2.0, and the Child and Family Follow-up Survey (CFFS) [14,15]. In addition, we translated the WHODAS 2.0 and the CFFS into Chinese, which were then back-translated into English. The needs assessment tools were also refined based on the ICF checklist and the ICF-CY and its reliability was examined in this step.

#### Step 6: A nationwide study

We collected nationwide data and compared them with those obtained from the old system. The sample was 7,329 persons with 16 types of disabilities of the old system, including 7098 adults and 231 children. For most subjects, one physician and one of the following professionals: physical therapists, occupational therapists, speech pathologists, social workers, or nurses, in every authorized hospital were enrolled to conduct the disability evaluations after being trained. After disability evaluation, a social worker conducted needs assessments with the individual, family, and caregivers. All the evaluations were conducted using face-to-face interviews with the individuals with disabilities or their families/caregivers and by directly testing the individuals with disabilities.

#### Step 7: verify the evaluation procedure

Health and social welfare government officials, health professionals, and hospital administrative staff met regularly to discuss the appropriate evaluation procedure. To assist the people with disabilities in obtaining social welfare services, several procedures were proposed to meet the different needs and characteristics of those individuals. An information system framework was established and all data were managed through this information system and tables containing crucial information were generated automatically to be monitored by the responsible authorities [16]. One dataset of the information system, including 7,098 disabled persons aged 18 years or older, was used to compare the differences between the old and the new system.

### Phase III (July 2012-March 2013): implementation of the new system and impact analysis

#### Step 8: collaboration and monitor the new system

At this stage, we regularly monitored the results of the new system by looking into the tables that were generated automatically every month by the information system [16]. Central and local government officials also met regularly to discuss issues and collaborate to solve problems. The data of 168,052 persons who applied for disability services were used to investigate the impact of the new system.

The central government of Taiwan also formed five counselling groups to assist local governments and authorized assessment hospitals since March 2013. These groups consisted of multidisciplinary experts, government officials, scholars, and representatives of non-government social welfare organizations. They are tasked to monitor the procedures and outcomes of the disability evaluations and provisional statuses of the social welfare services.

## Results

### Development of the measures of the core set for disability evaluation

**Figure 4:**
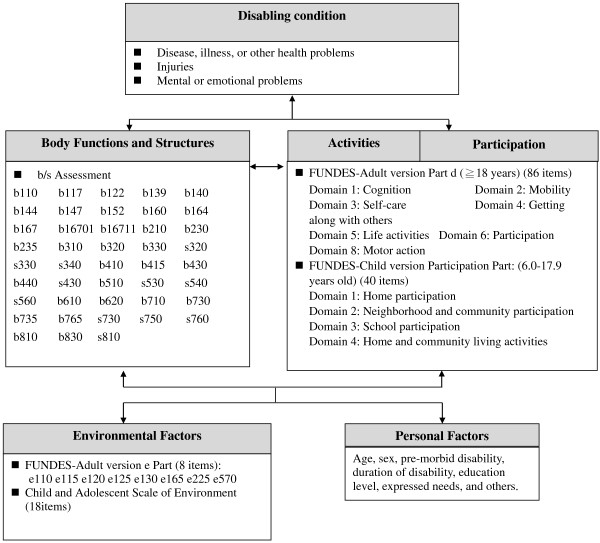
**ICF core set for disability evaluation.** Abbreviation: FUNDES: Functioning Scale of Disability Evaluation System, the adult version was modified from the second edition of the WHO Disability Assessment Schedule (WHODAS 2.0) and the child version was from the Child and Family Follow-up Survey (CFFS) with permission.

This study used Delphi technique to develop the b/s core set for the disability assessment [17-19]. (Figure 4) Forty-three b/s categories were included in the core set. Most items were second-level categories of the ICF and ICF-CY; however, to provide greater detail, certain items were fourth level categories.

For the ICF d components, we designed two scales to measure the functional status of two age groups. They were the FUNDES-adult for people aged 18 years and above, and the FUNDES-child version for children and youth (aged 6 to 17.9 years) [20,21]. The two FUNDES were designed based on the WHODAS 2.0 and the CFFS respectively.

The FUNDES-adult includes 94 items, with performance and capability dimensions in Domains 1 to 6 (cognition, mobility, self-care, getting along with others, life activities, and participation) and capability and capacity dimensions in Domain 8 (motor action). Domain 7 (environmental attributes) includes items that measure the perceived environmental barriers that people might encounter.

In the 2011 nationwide study, 7,098 adults with disabilities received the functional assessment, FUNDES-adult. The indicators of performance and capability for internal consistency were between 0.93 and 0.99 (Cronbach’s α). Factor analysis revealed a two-level hierarchical structure, the factor loadings of confirmatory factor analysis for the indicators of performance were between 0.8 and 0.89, and the capability was between 0.80 and 0.90 [21].

The FUNDES-child had 58 items, which covered four parts: physical and emotional health, participation, the Child and Adolescent Factors Inventory, and the Child and Adolescent Scale of Environment. The participation part includes items for measuring independence and frequency of participation and was derived from the Child and Adolescent Scale of Participation (CASP) [14,15,20]. Other parts measured health conditions, impairment of body functions (15 items) and environmental factors of the ICF-CY (18 items). The participation part had four domains: home participation (6 items), neighbourhood and community participation (4 items), school participation (5 items), and home and community living activities (HCLA; 5 items). The psychometric properties of the CASP-Traditional Chinese version have been found to be valid and reliable [20] (Figure 4).

### Development of evaluation tools for needs assessment

Evaluation tools for needs assessment were modified three times, and 81 items were included at the end. Each item has capacity and performance dimensions. People were also asked about their history of welfare applications, family support, and environmental factors. The assessment was conducted with face-to-face interviews with the persons or their families. Among the 7,098 persons in 2011, 6114 persons were used to examine the reliabilities of the needs assessment. The internal consistency of the needs assessment was between Cronbach’s α 0.87 and 0.99.

### Procedures for disability evaluation and welfare services

**Figure 5:**
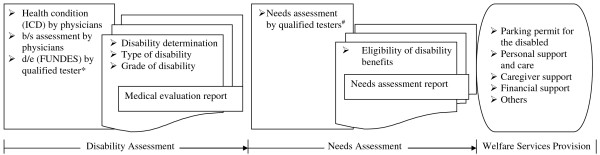
**National framework of disability evaluation and welfare services A national framework of disability evaluation and welfare services based on the WHO ICF.** Abbreviation: WHO: World Health Organization; ICF: International Classification of Functioning, Disability, and Health. *The tester is a professional who is qualified after receiving a full training course of Functional Scale of Disability Evaluation System (FUNDES). #The tester is a professional who is qualified after receiving a full training course of Needs assessment.

The procedures for disability evaluation and welfare services are depicted in Figure 5. In most situations, one physician gives the ICD-9-CM diagnosis codes and performs the b/s assessments, one qualified tester assesses the d/e components and the FUNDES. After the two assessments at the authorized hospital, a medical evaluation report with the information of disability determination, type of disability and grade of disability will be sent to the local social welfare bureau to arrange a needs assessment for that applicant. After the thorough evaluation of the needs assessment by another qualified tester, the eligibility for disability benefits is determined and finalized and the disability identification will be issued to the applicant, so that person’s needs for state support and services can be provided. There are several types of social welfare services, including: (1) parking permit for the disabled, (2) personal support and care (i.e. vocational rehabilitation, home care, residence/housing in community etc.), (3) caregiver support (i.e. temporary/respite and short-term care, training program for caregivers etc.), (4) financial support (i.e. disability pension, tax reduction etc.) (5) and others (i.e. assistive technology allowance etc.). Thus, the process for a disability evaluation requires at least three authorized specialists for each person with disabilities due to the multiple services. Qualified testers of FUNDES should be professionals who have experience of providing service to people with disabilities for at least 1 year in their field (i.e., physical therapy, occupational therapy, speech therapy, psychology, or social work) and have passed the required qualification tests. Between 2010 and 2012, we trained more than 6,000 FUNDES testers nationwide to perform the new disability evaluation in Taiwan [22].

### Differences between the old and new systems

To compare the differences of disability grades between the old and new systems, the data of 7,098 disabled persons aged 18 years or older were used in this study, 3,869 men (54.5%) and 3,229 women (45.5%) with an average age of 57.4 ± 17.6 and 60.1 ± 18.7 years, respectively. The causes of disability included visual impairment, hearing impairment, speech dysfunction, motor dysfunction, mental intellectual impairments, vital organ impairment, facial damage, dementia, autism, chronic mental disease, and rare disease. The three leading types of disability were motor dysfunction (28.5%), chronic intellectual impairments (26.2%), and hearing impairment (10.0%). The proportion of impairment types of this sample was similar to the national population with disabilities (*P* > .05). The results showed a 49.7% agreement of disability grades between the old and new systems.

### Impacts of the new system

A total of 168,052 persons applied for services through the new system, and 110,667 (65.9%) persons were eligible for disability benefits and received the needed social welfare services to enhance their social participation. Among them, 85,276 persons (77.1%) received basic social welfare support, 51,885 persons (46.9%) were issued parking permits for the disabled, 98,928 persons (89.4%) received financial support, 26,211 persons (23.7%) gained assistive technology allowances, 8,171 persons (7.4%) received caregiver support, and 8,691 persons (7.9%) received nursing care and rehabilitation services at home.

## Discussion

The ICF system was unanimously accepted by the World Health Assembly in 2001 [4]. It provides a robust classification system for collecting statistics on populations with disabilities. Although the ICF framework has been implemented in numerous countries, Few documentation currently exists on using the ICF system to classify people with disabilities on a nationwide basis [7,23-25]. This study demonstrated that disability eligibility system based on the ICF could provide a common language between disability evaluation, needs assessment and welfare services. This is crucial when social welfare resources are limited. By linking disability evaluations and needs assessments, we can reallocate social welfare resources and provide more resources to people in need. This study demonstrates a practical model for integrating the medical model with a biopsychosocial model based on the ICF.

The estimated prevalence of disability differs across nations. The reported prevalence ranges from less than 1% in Kenya, and 5% in South Africa and Bangladesh, to 20% in New Zealand [26,27]. In Taiwan, the disability prevalence was 4.7% in 2012 [3]. Differences in the estimated prevalence among countries may arise through several factors, including different cut-off values for eligibility for disability benefits, different methodologies and data collection, and variations in the quality of the study design. A vital factor that influences the reported prevalence rate is the purpose of disability evaluation. In Taiwan, the purpose of disability evaluation is mostly for the subsequent allocation of welfare services and allowances [28]. That’s why the prevalence rate of disability is lower than that of most the other countries.

The ICF model conceptualizes disability as arising from the interaction between the health condition of a person with his/her physical, cultural, and policy environments [29,30]. If the environmental factors were designed to accommodate the full range of human functioning, and were to incorporate appropriate services and support, people with functioning problem would not be “disabled” and would be able to participate fully in society. Services and supports are thus not only emphasized at the individual level (e.g., medical treatment) but also focused at the societal level [31]. Thus, the Taiwanese government selected the ICF as the preferred model to assist with the reformation of disability evaluation in the country [32].

In Taiwan, the disability assessment consists of 32 bodily functions, 11 body structures and 94 items of FUNDES for adult /58 items for child, respectively. All of these codes and items were adopted from the ICF checklist [33], ICF-CY, WHODAS 2.0, and CFFS. However, only five bodily functions and 15 activity participation codes are used to evaluate person eligibility for social security in Europe [34]. Whether too many categories for disability assessment were used in Taiwan needs to be further investigated.

In its old disability service system, Taiwan adopted a residual welfare model [35]. Every person with disabilities had the similar benefits from the government. For example, everyone had disability parking permits and assistive device allowances. Besides, in the old system, the government allocated the benefits mainly depending on the severity instead of on the basis of needs. Such support might be not able to enhance the participation of persons with disabilities. After reformation, with the new system, only 46.9% had disabled parking permits and 23.7% gained assistive device allowances. Besides, the government provided services according to results in the functional and needs assessments.

Based on Taiwan’s experiences of disability evaluation using the ICF, the following guiding principles are suggested for countries that might use ICF in their disability evaluation. For hospitals and other medical providers, a multidisciplinary team approach will be required to complete the evaluations, and the number of testers and rooms for testing must be expanded. For local government, the budget for disability assessments should be increased than the traditional medical model and more collaboration and information sharing among different sectors (such as health, social welfare and labor) and disability groups to resolve case disputes. For the central government, quality of professionals for the disability evaluation should be regulated, and that the diverse resources and delivery system should be organized effectively to meet the various needs of people with disabilities, and the results of the disability evaluation should be integrated with other social welfare system, such as long-term care or medical care to decrease redundant evaluations. For people with disabilities and their families, some might be affected by a change in the new system, such as pension might be less, however, the services related to social participation would be enhanced.

### Limitations

The final determination of the disability grade was obtained from a summary of assessment. However, no empirical evidence or nationwide data currently exist for justifying the use of certain cut-off values for disability evaluation. The validity of the cut-off values to determine the disability grade has yet to be established and needs further research. Instruments and methods require further testing and validation.

## Conclusions

This study demonstrated that disability evaluation system based on the ICF could provide a common language between disability assessment, needs assessment and provision of welfare services. We implemented a new system to assess a person’s eligibility for disability benefits at a national level. However, the proposed procedure and tools require additional testing and validation.

## Abbreviations

ICF: International classification of functioning, disability, and health; WHO: World Health Organization; CRPD: Convention on the rights of persons with disabilities; WHODAS 2.0: WHO disability assessment schedule 2.0 version; CFFS: Child and family follow-up survey; WAIS-III: Wechsler adult intelligence scale third edition; FUNDES-adult: Functioning scale of disability evaluation system -adult version; FUNDES-child: Functioning scale of disability evaluation system- child version; CASP: Child and adolescent scale of participation; HCLA: Home and community living activities.

## Competing interests

The authors declare that they have no competing interests.

## Authors’ contributions

WTC and THL participated in the design of the study. CFY, SWT and WCC contributed to acquisition of the data. HFL, KHC and YHW perform the statistical analysis and interpretation of the data. WTC, CFY, SWT, HFL and THL drafted the manuscript. KHC and YHW revised the manuscript critically. All authors read and approved the final manuscript.

## Pre-publication history

The pre-publication history for this paper can be accessed here:

http://www.biomedcentral.com/1472-6963/13/416/prepub
